# 

**Published:** 1903-08-15

**Authors:** 


### NOTICE TO CORRESPONDENTS.

All MSS., Letters, Books for Review, and other matters intended for the Editor should be addressed The Editor, "The Hospital," The Hospital Buildings, 28 & 29 Southampton Street, Strand, W.C.

The Editor cannot undertake to return rejected MS., even when accompanied by stamped directed envelope.

**Notice to Advertisers and Subscribers.**

All Advertisements, Orders for Copies of The Hospital, and Business Communications should be addressed to **THE MANAGER, (Not to the Editor)**, The Hospital, 28 & 29 Southampton Street, Strand, London, W.C.

The Cost of Subscription, post free, is as follows.—Per week, 3½d.; per quarter, 3s. 9d.; per half-year, 7s. 6d.; per annum, 15s. Foreign and Colonial:—Per annum, 19s. 6d., payable, in all cases, in advance.

**NOTICE.—Vol. XXXIII. of "The Hospital" (October 1902 to March 1903) in handsome cloth binding, will shortly be on sale at the office of this Journal, price 7s. 6d. Cases for binding the Numbers contained in this Volume 1s. 6d. Index to Vol. XXXIII., 2½d., post free.**

**The monthly part for August is now ready. Price 1s., by post 1s. 3d.**

### Books Received.

Rebman, Limited.

"Some Practical Points in the Diagnosis and Treatment of Gonorrhœa in the Male." By H. Oppenheimer.

Baillière, Tindall and Cox.

"Comparative Odontology." By A. S. Underwood.

Kegan Paul and Co.

"Bradshaw's Bathing Places and Climatic Health Resorts."

Walter Hill.

"The Holidays 1903. Where to go and what to see."

### THE STUDENT OF MEDICINE.

### APPOINTMENTS.

**Victor George Alexander**, M.B., B.Sc.Edin., Assistant District Surgeon for Johannesburg. **A. E. Boycott**, M.A., M.B., B.Ch., B.Sc.Oxon., Gordon Lecturer in Experimental Pathology at Guy's Hospital Medical School. **F. L. Harman Brown**, M.B., C.M.Edin., to be Honorary Medical Officer to the Coventry and Warwickshire Hospital. **William Brodie Brown**, M.B., C.M., Medical Officer and Public Vaccinator for the Western Division of the Parish of Birse. **Walter Ponsonby Cockle**, B.A., M.D., B.Ch.Dub., Anæsthetist to the Metropolitan Ear, Nose, and Throat Hospital, Grafton Street, W. **Archibald Cuff**, B.A., M.B., B.C.Cantab., F.R.C.S.Eng., Surgeon to the Royal Infirmary, Sheffield. **John H. P. Fraser**, M.B., M.R.C.S., Medical Referee under the Workmen's Compensation Acts for Southampton, Winchester, and Romsey, in County Court Circuit No. 51. **T. Gordon Kelly**, B.A., M.D., D.P.H., Lond., reappointed Medical Officer of Health for Market Bosworth Rural District. **F. W. Marlow**, M.D., C.M.Trin.Tor., L.R.C.P.Lond., F.R.C.S.Eng., Assistant Surgeon to St. Michael's Hospital, Toronto. **John Pardoe**, M.B. Aberd., F.R.C.S.Eng., Assistant Surgeon to St. Peter's Hospital, Covent Garden. **F. J. Poynton**, M.D., F.R.C.P.Lond., Assistant Physician to University College Hospital. **Lilian G. Simpson**, M.D.Brux., L.R.C.P.&S.Edin., Assistant Medical Officer to the Camberwell Infirmary, Camberwell, S.E. **J. W. Thomson Walker**, M.B., C.M.Edin., F.R.C.S.Eng., Assistant Surgeon to St. Peter's Hospital, Henrietta Street, W.C. **E. S. Worrall**, L.R.C.P., M.R.C.S., Medical Officer in charge of the Radiographic Department, Victoria Hospital for Children, Tite Street, Chelsea.

### "The Hospital" Institutional Library.

"Hospitals and Asylums of the World." 4 Vols., with a Portfolio of Plans, complete £12 12s.
"Burdett's Hospitals and Charities, 1903." 5s. net.
"Burdett's Official Nursing Directory, 1903." 3s. net.
"Cottage Hospitals, General, Fever, and Convalescent." 10s. 6d.
"Uniform System of Accounts for Hospitals and Public Institutions." New Edition. 4s. net.
"Hospital Expenditure: The Commissariat." 2s. 6d.
"A Legal Handbook for the Use of Hospital Authorities." 2s. 6d.
"The Cottage Hospital Case Book or Register of Patients." 10s. and 12s. 6d.
All these are published by the SCIENTIFIC PRESS, Ltd., and may be obtained through any bookseller, or direct from the publishers, 28 and 29 Southampton Street, Strand, London, W.C.

### The Best and most up-to-date.

Uniform with the Nurses' Dictionary.

The work of a Practical Teacher.

Profusely Illustrated.

*JUST PUBLISHED.*

A

### PRACTICAL GUIDE

TO

### SURGICAL BANDAGING

AND

### DRESSINGS.

By

### Wm. JOHNSON SMITH, F.R.C.S.

**Principal Medical Officer Seamen's Hospital, Greenwich.**

Simple Explanations of Scientific Principles.

The Surgical Nurse's Pocket Companion.

Price 2/- Post free.

*Obtainable of all Booksellers, published by*

THE SCIENTIFIC PRESS, LIMITED, 28 & 29 Southampton Street, Strand, London,

*who will send their latest Catalogue of Nursing Text-books post free to any Nurse on application.*

